# *Cuscuta australis* Parasitism-Induced Changes in the Proteome and Photosynthetic Parameters of *Arabidopsis thaliana*

**DOI:** 10.3390/plants11212904

**Published:** 2022-10-28

**Authors:** Lyuben Zagorchev, Zhaokui Du, Yongbin Shi, Denitsa Teofanova, Junmin Li

**Affiliations:** 1 Zhejiang Provincial Key Laboratory of Plant Evolutionary Ecology and Conservation, Taizhou University, Taizhou 318000, China; 2 Department of Biochemistry, Faculty of Biology, Sofia University “St. Kliment Ohridski”, 8 Dragan Tsankov blvd., 1164 Sofia, Bulgaria

**Keywords:** dodders, parasitic plants, proteomics, photosynthesis

## Abstract

*Cuscuta australis* is a widely distributed stem parasitic plant, infecting a variety of host plants. Its parasitism has a negative effect on the hosts, mainly due to the exhaustion of nutrients, thus negatively affecting the growth and development. However, recent studies indicated that the effect of parasitism may extend beyond the simple extraction of organic compounds, water, and minerals. In the present study, the model plant *Arabidopsis thaliana* was used as a host for *Cuscuta australis*, to study the effect of the parasite on the photosynthetic parameters and the proteome after short-term infection. To test this, a highly sensitive portable photosynthesis system and gel-based MS/MS proteomics were employed. It was found that the parasite has a dramatic negative effect on the photosynthetic ability of the host, as well as causing the up-regulation of stress-related proteins. Simultaneously, proteins involved in both decreased permeability and loosening of the cell wall of the host were found to be up-regulated.

## 1. Introduction

Dodders (*Cuscuta* spp. L., Convolvulaceae, Solanales) are prominent parasitic angiosperms infecting a wide range of predominantly eudicotyledonous host plants [1]. Dodders are stem parasites, e.g., infecting the aerial organs, either stems or leaves, of the hosts. They lack or have limited photosynthesis [2] and lose soil contact after the first successful infection. Instead of autotrophic carbon fixation, they acquire organic as well as mineral nutrients and water from their hosts by establishing a physiological xylem/phloem link, known as haustoria [3]. By doing this, they largely exhaust their host plants and exhibit a negative impact on their growth and development [4].

Most of the current research on dodders is focused on prevention and management strategies due to their potential to be agricultural pests. Several species, indeed, are considered a serious threat to contemporary agriculture and cause significant yield reduction in over 50 economically important crop plants [5]. Although generally regarded as harmful, more and more reports suggest the important role of dodders as biodiversity regulators and an important part of healthy plant societies. They are known to selectively forage on certain species under changing environmental conditions, thus benefiting the growth of other species [6]. Recently it was shown that native *Cuscuta* spp. might be key players in reducing the spreading of invasive species [7]. In China, both *C. australis* R. Br. and *C. chinensis* Lam. were proven as efficient regulators of invasions of several introduced weeds, most notably *Mikania micrantha* Kunth. [8,9].

Although dodders were extensively studied for their agricultural and ecological impact, knowledge of the host–parasite interactions is still fragmented. A typical dodder lifecycle includes germination, host localization, coiling (also twining), and haustoria formation through penetration to vascular elements of the host, formation of secondary stem and infectious sites, ending with flowering, seed formation, and dispersal [1]. Host localization is believed to involve both light [10] and chemical [11] signal perception. Formation of haustoria was recently reviewed by Shimizu and Aoki [12] and divided into three distinct phases. First, a tight adhesion is achieved through the secretion of adhesive substances and elongation of *Cuscuta* stem cells toward the host tissue. There are reports, that both pectic substances [13] and arabinogalactan proteins (AGPs) are responsible for the adhesion [14]. The intrusive phase involves differentiation of various cell types, forming searching hyphae and penetrating the host tissue by pushing the host’s cells aside [13]. Various cell wall hydrolyzing and modifying enzymes are also involved in this process in order to loosen host cells’ walls and facilitate the process [15,16]. Finally, during the conductive phase, the searching hyphae differentiate into xylem and establish a xylem (and probably phloem) bridge with the host [12].

Further growth of the parasite results in continuous host exhaustion of both assimilated carbon and nitrogen, and thus, significant host growth restriction [4]. While Jeschke and Hilpert [17] reported increased photosynthetic activity in the host as a compensatory mechanism, more recent literature reported a decrease in photosynthetic parameters [18,19]. We also recently demonstrated that at least the light phase of photosynthesis may be inhibited even in *Cuscuta* association with poor hosts, where the growth of the parasite is not abundant [20]. This is an important question as it seems that, even at a slow growth rate, the parasite could inhibit the overall performance of the affected hosts.

Accumulation of new knowledge about the molecular mechanisms of dodder–host plants interaction is crucial from a fundamental point of view, but also important for the development of new strategies for control and eradication in agriculture. Such efforts are impeded by both the long seed longevity and continuous germination over the years [10], but most importantly by the difficulties during post-attachment control. By establishing a physiological link with the host plant, dodders are difficult to remove without harm to their hosts and there is a need for highly selective herbicides, affecting only the parasite and no other plants [21]. Elucidation of the host’s response to dodder infection could also help the selection of resistant cultivars [22,23].

Recent reports demonstrated the dodder-specific response of at least some host species, accomplished by specific receptors and response proteins [24], as well as Jasmonic acid and Salicylic acid response pathways [25]. Research on these aspects of dodder–host interactions is still scarce, inconclusive, and further obstructed by the apparent interspecific and intraspecific variations. It was not until recently that dodder research was focused on the use of *Arabidopsis thaliana* (L.) Heynh. as a model host plant [26]. In the present study, we performed combined measurement of photosynthetic parameters with gel-based proteomics study on the effect of *C. australis* parasitism on *A. thaliana*. The aim was to assess both the photosynthetic performance, which was proposed to be significantly affected by parasitism, and differentially abundant proteins in the host, separately, in the affected stem and the relatively distant leaves.

## 2. Results

### 2.1. Effect of Parasitism on Photosynthetic Parameters

**Figure 1:**
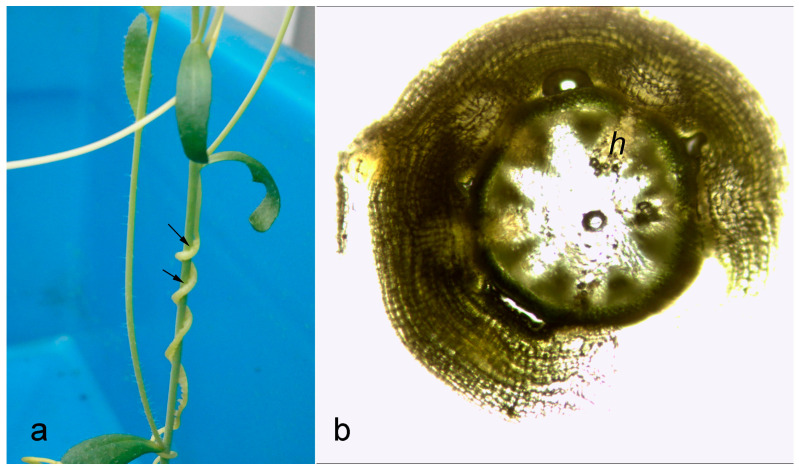
Point of infection of *Arabidopsis thaliana* by *Cuscuta australis* (**a**) with arrows, showing the site of haustoria formation after 24 h and microscopic cross-section of haustoria (h) after 72 h (**b**).

Attachment and haustoria formation were macroscopically visible at the 24th hour in most cases (Figure 1a) and were further confirmed microscopically (Figure 1b). Despite the fact that there were slight differences in terms of time to attachment, further analyses were performed on infected Arabidopsis plants with the most uniform pattern of infection. *Cuscuta australis* parasitism decreased most of the photosynthetic parameters in the host, *A. thaliana*, within 72 h of infection (Table 1). A notable drop in relative chlorophyll content—almost two-fold from 17.5 to 8.8—as well as a significant decrease in photosynthetic rate (*P*_n_), stomatal conductance (*g*_s_), and transpiration rate (*T*_s_) was observed. The change in intercellular CO_2_ (*C*_i_) was not significant but followed the pattern of decrease.

### 2.2. Quantitative Analysis of Protein Spots on 2D Gels

**Figure 2:**
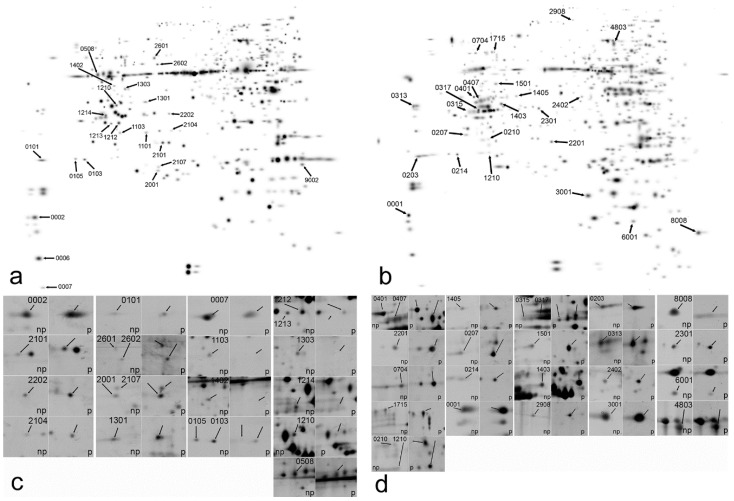
PDQuest master gel images of 2D-electrophoregrams of Arabidopsis leaves (**a**) and stem (**b**) and the respective differentially regulated protein spots (**c**,**d**). Arabidopsis was either parasitized (p) or non-parasitized (np) by *Cuscuta australis*. Protein spot numbers correspond to the assigned by PDQuest.

**Figure 3:**
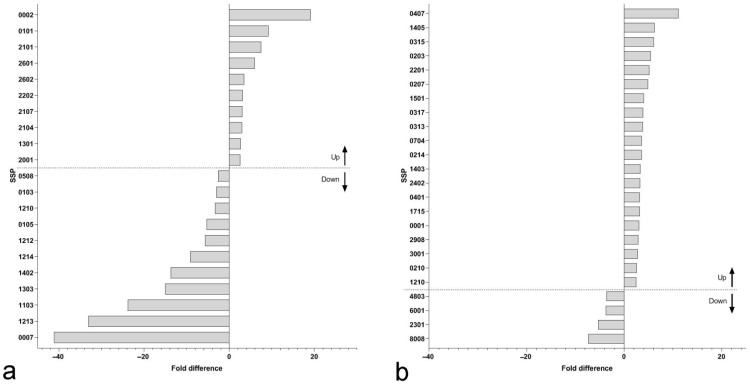
Differentially regulated protein spots, shown as SSP numbers, assigned by PDQuest with 2.5-fold difference in leaves (**a**) and stem (**b**) of *Cuscuta australis*—parasitized *Arabidopsis thaliana*.

Differential protein abundance was assessed by two-dimensional polyacrylamide gel electrophoresis, separately in stem and leaves of the host plants. Images were analyzed using PDQuest 8.0 (Bio-Rad, Hercules, CA, USA) Software by deriving a master gel image (Figure 2a,b). Original images are provided as (Supplementary Figures S1–S4). Only protein spots with 2.5-fold difference between non-parasitized and parasitized samples, statistically significant at *p* < 0.05, Student’s *t*-test (Figure 2c,d) were considered for further analysis. A total of 21 proteins spots in leaves and 24 protein spots in the stem met the above criteria (Figure 3). Of them, the proportion of up-regulated to down-regulated protein spots were almost equal in leaves (Figure 3a), while in the stem (Figure 3b) the number of up-regulated protein spots was substantially higher than down-regulated (only four). The fold difference in leaves was also higher, reaching 40-fold down-regulation for SSP 0007 and above 20-fold for SSP 1213 and 1103 (Figure 2c and Figure 3a). The fold difference in stem was much lower for all of the protein spots (Figure 3b). All protein spots are indicated by their SSP number, automatically assigned by PDQuest 8.0 Software.

### 2.3. Protein MS/MS Identification

A total of five protein spots in the leaves and two protein spots in the stem failed to be identified (Figure 3)—protein score, lower than the identity or extensive homology score at *p* < 0.05. The additional broad database search (Viridiplantae) aimed to find any possible proteins of *Cuscuta* origin, as such are known to be transferred into hosts [27]. However, no such were found. Out of the identified protein spots (Table 2 and Table 3), one protein was found in a total of four down-regulated spots in leaves glyceraldehyde-3-phosphate dehydrogenase (SSP 1210, 1212, 1213, and 1303). One protein was found in two up-regulated spots in stem Atmp 24.1 glutathione S transferase (SSP 2201 and 3001), but also two isoforms of aspartate aminotransferase were found in two up-regulated spots (SSP 0315 and 1403). Chloroplastic transketolase 1 was found in up-regulated spots in both leaves and stem (SSP 2601 and 1715, respectively) and chloroplastic phosphoribulokinase was found in down-regulated spots in both leaves and stem (SSP 0508 and 8008, respectively). Finally, a member of the glycine cleavage T-protein family was found in a down-regulated spot in leaves (SSP 0105) and up-regulated spot in the stem (SSP 0407).

According to the annotation to gene ontology (GO) biological process, proteins, associated with a variety of stress, including biotic (response to bacterial pathogens) and abiotic (salinity, cold, heavy metals) were equally distributed between both up-regulated and down-regulated protein spots (Table 2 and Table 3). Except for down-regulated protein spots in the stem, the stress-related proteins were not the predominant fraction in either of the identified proteins. A substantial amount, however, is associated with either photosynthesis or carbohydrate metabolism.

## 3. Discussion

The pronounced effect of *C. australis* parasitism on the host’s photosynthetic parameters is consistent with previously reported results for the *C. campestris—M. micrantha* association [18], who reported a significant decrease in *P*_n_ after 18 days of infection and *C. australis—Bidens pilosa* [28,29]. Chen [30] suggested the involvement of abscisic acid (ABA) in the suppression of stomatal conductance and transpiration rate, hence the net photosynthetic rate. Despite differences in the host response in terms of time needed for the adverse effect of *Cuscuta* infection on photosynthesis to occur, which might be host-specific and dependent on the parasite size, an overall decrease in photosynthetic parameters seems like a common response. Considering the light reactions, we also recently demonstrated a certain negative effect of *C. campestris* parasitism on the semi-compatible host *Ipomoea tricolor* [20], suggesting a dramatic effect on the host plant even when the parasite is not developing successfully. Overall, it seems that suppression of photosynthesis in the early stages of *Cuscuta* parasitism is a global stress response, similar or sharing common signal mechanisms to other abiotic, mainly drought [31] and biotic [32] stresses.

The decrease in CO_2_ assimilation could be further explained by the down-regulation of at least the large RuBisCO subunit (Table 3), but also the down-regulation of the chloroplastic Glyceraldehyde-3-phosphate dehydrogenase and Phosphoribulokinase (Table 3), both of which are involved in the regeneration of ribulose-1,5-bisphosphate. Accordingly, down-regulation leads to a reduction in CO_2_ assimilation, otherwise designated as “Calvin-Benson Cycle slow down” [33,34]. In all cases, such down-regulation of CO_2_ assimilation would largely contribute to the growth inhibition of parasitized hosts, reported previously [18,35] in addition to the reduction in organic nutrients [4]. Furthermore, many of the differentially regulated proteins in the present study are related to the modulation of the carbohydrate or amino acid metabolism and according to GO biological processes are related to the response to different kind of stresses. In contrast, we also found up-regulation of the small RuBisCO subunit, several thylakoid complex proteins—Oxygen-evolving enhancer proteins (PSBO2), and photosystem II subunit P-1 (Table 2). The small RuBisCO subunit is encoded in nuclear DNA and synthesized in the cytoplasm, then it is transferred into the chloroplast [36]. The up-regulation of the small RuBisCO subunit indicated that the compensatory reaction of the nuclear gene to decreased accumulation of photosynthates would be quicker than genes encoded in chloroplast DNA, such as the large RuBisCO subunit gene. PsbO proteins in Arabidopsis are encoded by two genes, *psbO1* and *psbO2* [37]. A low level of PSBO2 would limit the photosynthetic activity [37] and the function of PBSO2 showed under high light stress. In this study, Arabidopsis plants were grown under natural sunlight conditions in the greenhouse for successful infection of *Cuscuta*. The up-regulation of PSBO2 might be due to the high light stress response.

Overall, the host’s response to *Cuscuta* infection is believed to trigger Jasmonic acid and Salicylic acid defense pathways [25] and up-regulation of numerous pathogen-response related genes and proteins was previously reported. Borsics and Lados [38] reported up-regulation of *PPRG2* in dodder-infected alfalfa, encoding a homologous to pathogenesis-related (PR-10) protein family product. In *Mikania micrantha*, infected by *C. campestris*, up-regulation of a homologous to chitinase gene, *Mmchi1*, was reported [39]. In our study glutathione S-Transferase Atpm24.1, commonly involved in response to pathogenic fungi, bacteria, and viruses, mainly governing the antioxidant response [40] was found to be up-regulated in the stem of infected plants. More interestingly, it was found that S-adenosylmethionine synthase 4 was up-regulated in leaves and Pectin methyl esterase 3 in the stem. The former is involved in the synthesis of S-adenosylmethionine, an important methyl donor for methylation of lignin precursors for lignin synthesis [41]. As such, it is expected to be involved in the lignification and reinforcement of the cell wall, a common defense mechanism and systemic response against different pathogens [42]. Lignin-based resistance to *C. campestris* in the tomato plant was established in a particular cultivar, governed by up-regulation of numerous related genes [22]. The up-regulation of Pectin methyl esterase, especially in the stem of the host plants, where infection with the dodder mainly occur is probably involved in the de-esterification of pectic polysaccharides, which in turn facilitates pectin hydrolyses and penetration of the parasite [43]. It is yet to be concluded whether this is a mechanism by which the parasite manipulates the metabolism of the host to increase susceptibility, but it was reported that pectinolytic enzymes are essential in host tissue penetration during haustoria formation [43].

The possibility that some of the up-regulated proteins in the parasitized group are of *Cuscuta* origin was taken into consideration because of several studies reporting extensive protein exchange between the parasite and the host [27,44], including translation of transferred mRNAs in the host [45]. Liu et al. [27] reported more than 1000 dodder proteins, translocated to the infected *Arabidopsis* stems. Interestingly, most of the up-regulated and none of the down-regulated proteins in our study were also found to be dodder-to-*Arabidopsis* mobile proteins [27].

## 4. Materials and Methods

### 4.1. Plant Material and Growing Conditions

Seeds of *Arabidopsis thaliana* ecotype Columbia-0 (Col-0) were kindly provided by Dr Zhongnan Yang (Shanghai Normal University, Shanghai, China). Arabidopsis seeds were surface sterilized using 70% (*v*/*v*) ethanol for 10 min, then washed three times with sterile water and stratified for 48 h at 4 °C in the dark. The treated seeds were sown evenly in vermiculite: peat substrate: perlite (16:3:1) mixture and germinated in a growth chamber under 16 h light/8 h dark photoperiod, 150 μmol m^−2^ s^−1^ white light intensity, 22 °C, 70% relative humidity. Two-week-old healthy seedlings were transferred to a plastic tray (one seedling per tray), containing a controlled-release fertilizer (Osmocote^®^, Scotts International B.V., Harderwijk, The Netherlands), and grown under the same conditions. Twenty plants were chosen when the height reached approximately 5 cm in a growth chamber, then separated into the control group and parasitized group (each group had 10 plants) and grown for further 5 days.

Fifteen cm long stems of *C. australis* were collected from a field population at Taizhou University, Linhai City, Zhejiang Province, China. The parasite’s stem was coiled around the inflorescence stem (the upper one-third, approximately 5 cm from the rosette leaves) of *A. thaliana* (one parasite per host) in a counter-clockwise direction and plants were grown under natural sunlight conditions in the greenhouse for infection—usually, haustoria are formed after 24 h. After 72 h, a total of six plants from the parasitized group with the most uniform *Cuscuta* infection and six plants from the control group, respectively, were chosen for photosynthetic measurements and protein analyses.

### 4.2. Photosynthesis Measurements

Relative chlorophyll content was measured using CCM-200 chlorophyll content meter (OPTI-SCIENCES, Hudson, NH, USA) on fully expanded 5th rosette leaves of Arabidopsis, one leaf per plant. Photosynthetic rate (*P*_n_), stomatal conductance (*g*_s_), the concentration of intercellular CO_2_ (*C*_i_), and transpiration rate (*T*_s_) were measured using a portable photosynthesis system Li-6400 (LI-COR, Lincoln, NE, USA) according to the manufacturer’s instructions on fully expanded rosette leaves. Gas exchange measurements were determined under 25 °C, 1200 μmol m^−2^ s^−1^ photons, 400 μmol mol^−1^ CO_2_ concentration and 70% relative humidity.

### 4.3. Protein Extraction and Quantification

For proteomics analyses, Arabidopsis inflorescence stem (2 cm up and down the site of dodder parasitism) and leaves (5th rosette, two leaves per plant) were studied separately after 72 h of infection. Total protein was extracted from the control and parasitized tissues of Arabidopsis as previously described [46]. Two grams of fresh plant material were powder-grounded in liquid nitrogen, dissolved in 20 mL cold acetone, containing 10% (*w*/*v*) trichloroacetic acid (TCA), and proteins were precipitated for 1 h at −20 °C. The samples were centrifuged at 15,000 *g* for 15 min at 4 °C. The procedure was repeated with TCA-free cold acetone, then the pellets were dried by vacuum freeze dryer and dissolved in protein lysis buffer, containing 9 M Urea, 4% (*w*/*v*) CHAPS, 1% (*w*/*v*) DTT, 1% (*v*/*v*) IPG buffer (pH 4–7, GE Healthcare, Danderyd, Sweden) and vortexed at 30 °C for 1 h. The protein concentrations were determined by Bradford’s method [47]. If not immediately used for isoelectric focusing separation, proteins were stored at −80 °C.

### 4.4. Two-Dimensional Gel Electrophoresis

Equal amounts of protein (100 µg) were separated on Immobiline Dry Strip Gels (pH = 4–7, 24 cm, GE Healthcare, Danderyd, Sweden). Isoelectric focusing (IEF) was performed according to the producer’s recommendations at 20 °C as follows: 250 V for 1 h, 1000 V for 2 h, on a linear gradient from 1000 V to 10,000 V for 1 h, 10,000 V for 1 h. The strips were then incubated under constant gentle shaking in equilibration buffer I (6M urea, 30% *v*/*v* glycerol, 2% *w*/*v* SDS, 50 mM Tris-HCl pH 8.8, 1% *w*/*v* DTT) and buffer II, in which DTT was replaced with 2.5% *w*/*v* Iodoacetamide, for 15 min each. Twelve percent T SDS polyacrylamide gels were used for the second-dimension electrophoresis and the gels were stained with Coomassie Brilliant Blue (CBB) G-250 according to the procedure described by Neuhoff [48].

### 4.5. Image Analysis and Mass Spectrometric Identification

The stained gels (in triplicates, each representing an individual plant) were scanned on Image Scanner (GE Healthcare, Waukesha, WI, USA) at a resolution of 300 dots per inch and the images were analyzed using PDQuest 2-D analysis software 8.0 (Bio-Rad, Hercules, CA, USA), following the steps of spot detection, volumetric quantification and matching. Protein spots, identified to show 2.5-fold higher or lower difference (*n* = 3, *p* < 0.05, Student’s *t*-test) in intensity between control and parasitized Arabidopsis plants were selected as the differentially abundant proteins, designated up-regulated and down-regulated, respectively, and extracted for MS analysis. Selected protein spots were excised from 2-DE gels, de-stained, dehydrated, and digested with trypsin according to the procedure of Shevchenko [49]. Peptide MS and MS/MS were performed on an ABI 5800 MALDI-TOF-TOF plus mass spectrometer (Applied Biosystems, Foster City, CA, USA).

Both the MS and MS/MS data were integrated and processed using the GPS Explorer V3.6 software (Applied Biosystems, Foster City, CA, USA) with default parameters. Based on combined MS and MS/MS spectra, the protein identification was performed using the MASCOT V2.1 search engine (Matrix Science, London, UK), with the following search parameters: NCBIprot (also performed against SwissProt database) thale cress (*Arabidopsis thaliana*) database (repeated against green plants, Viridiplantae), trypsin as the digestion enzyme, one missed cleavage site; variable modifications: Oxidation (M), Acetyl (Protein N-term), Deamidated (N and Q) and Dioxidation (W); fixed modifications: Carbamidomethyl (C), and a mass deviation of less than 100 ppm. Statistically significant search scores (>95% confidence, equivalent to MASCOT expect value *p* < 0.05) were chosen [50]. All confirmed proteins—MASCOT *p* < 0.05 were searched in UniProt database and GO annotated for localization and biological process.

## 5. Conclusions

In conclusion, it was shown that *Cuscuta australis* parasitism negatively affected most of the photosynthetic parameters of the host plants. The differential abundance of proteins, however, could not entirely explain such effect. Nevertheless, multiple proteins related to the carbohydrate metabolism were differentially regulated, as well as proteins with known function in abiotic and biotic stress response and defense mechanisms. Notably, S-adenosylmethionine synthase 4 and Pectin methyl esterase 3, involved in cell wall modifications and the parasite–host interaction, are up-regulated in stem and leaves, respectively. Accumulation of data on proteins involved in plant-to-plant interaction could further elucidate the mechanisms of haustoria formation in *Cuscuta* spp. and allow us to identify individual players in the hosts’ defense with a putative role in at least partial resistance to infection.

## Figures and Tables

**Table 1:** Effect of parasitism on the chlorophyll content and photosynthetic characteristics of the host. Data were shown as mean ± standard errors (*n* = 6). Different small letters indicate significant (Student’s *t*-test) differences between parasitized and non-parasitized treatments.

Traits	Non-Parasitized	Parasitized
Photosynthetic rate (*P*_n_)/(μmol CO_2_ m^−2^ s^−1^)	**3.61 ± 0.73^a^**	2.71 ± 0.33^b^
Stomatal conductance (*g*_s_)/(μmol H_2_O m^−2^ s^−1^)	**0.19 ± 0.05^a^**	0.12 ± 0.01^b^
Concentration of intercellular CO_2_ (*C*_i_) /(μmol CO_2_ m^−2^ s^−1^)	305.66 ± 1.34^a^	301.58 ± 6.23_a_
Transpiration rate (*T*_r_)/(μmol CO_2_ m^−2^ s^−1^)	**3.30 ± 0.74^a^**	2.31 ± 0.097^b^
Relative chlorophyll content	**17.51 ± 2.06^a^**	8.79 ± 1.96^b^

**Table 2:** MS/MS identified protein spots, up-regulated in leaves and stem of *Cuscuta australis*—parasitized *Arabidopsis thaliana*. SSP is the standard spot number.

SSP	Mascot Score	UniProt Accession	Protein Identity	GO Localization	GO Biological Process
**Leaves**					
0002	98	Q41932	Oxygen-evolving enhancer protein 3-2, chloroplastic	chloroplast thylakoid membrane	photosynthetic electron transport chain
0101	69	Q9LUT2	S-adenosylmethionine synthase 4	cytoplasm	lignin biosynthetic process response to cold stress
2101	54	Q9SAJ4	Phosphoglycerate kinase 3, cytosolic	Cytoplasm	gluconeogenesis
2601	175	Q8RWV0	Transketolase-1, chloroplastic	Chloroplast	Pentose-phosphate shunt Response to cadmium ions/salt stress
2602	53	P10795	Ribulose bisphosphate carboxylase small chain 1A, chloroplastic	Chloroplast	chloroplast ribulose bisphosphate carboxylase complex assembly response to cold
2107	130	O49344	Putative oxygen-evolving enhancer protein 2-2	Chloroplast	photosynthesis
1301	212	Q42560	Aconitate hydratase 1	Mitochondrion Cytoplasm	citrate metabolic process response to salt stress
**Stem**					
0407	269	O65396	Glycine cleavage T-protein family	Mitochondrion	glycine decarboxylation via glycine cleavage system response to cadmium ion
1405	229	B9DHX4	Malate dehydrogenase		carbohydrate metabolic process
0315	194	Q56YR4	aspartate aminotransferase	Multiple	Biosynthetic process
0203	229	A0A178UUR9	VDAC2	Mitochondrial outer membrane	voltage-gated anion channel
2201	169	Q8LC43	Atpm24.1 glutathione S transferase	Cytosol/ER	response to bacteria response to abiotic stress
1501	49	A0A178VU56	Succinate-CoA ligase [ADP-forming] subunit beta	Mitochondrion	tricarboxylic acid cycle
0317	352	Q9FWA3	6-phosphogluconate dehydrogenase family protein	Cytosol/Peroxisome	D-gluconate metabolic process response to salt stress
0313	49	A0A178VDL9	Pectin methyl esterase 3	Cell Wall	Cell wall modification
0704	550	O50008	methionine synthase	Cytoplasm	response to cadmium ion response to salt stress
1403	551	P46645	aspartate aminotransferase 2	Cytoplasm	2-oxoglutarate metabolic process
2402	191	Q944G9	Fructose-bisphosphate aldolase 2	Chloroplast stroma	gluconeogenesis response to abscisic acid/response to cadmium ion
0401	170	Q96533	glutathione-dependent formaldehyde dehydrogenase	Cytoplasm	ethanol oxidation
1715	197	Q8RWV0	Transketolase-1	chloroplast stroma	pentose-phosphate shunt response to cadmium ion/response to salt stress
0001	111	A0A178VBH5	PSBO2	Chloroplast thylakoid	photosystem II assembly
2908	334	Q93ZF2	putative 2,3-bisphosphoglycerate-independent phosphoglycerate mutase	Cytoplasm	glucose catabolic process
3001	168	Q8LC43	Atpm24.1 glutathione S transferase	Cytosol/ER	response to bacteria response to abiotic stress
0210	76	O24616	Proteasome subunit alpha type-7-B	Nucleus/Cytoplasm	proteasomal protein catabolic process
1210	127	Q42029	photosystem II subunit P-1	chloroplast thylakoid	Photosynthesis defense response to bacterium

**Table 3:** MS/MS identified protein spots, down-regulated in leaves and stem of *Cuscuta australis*—parasitized *Arabidopsis thaliana*. SSP is the standard spot number.

SSP	Mascot Score	UniProt Accession	Protein Identity	GO Localization	GO Biological Process
**Leaves**					
1213	259	A0A178VKK2	Glyceraldehyde-3-phosphate dehydrogenase		glucose metabolic process
1103	138	A0A0K1CVP8	Ribulose bisphosphate carboxylase large chain	Chloroplast	Photorespiration
1303	232	A0A178VKK2	Glyceraldehyde 3-phosphate dehydrogenase A subunit		glucose metabolic process
1402	138	P22954	dnaK-type molecular chaperone hsc70.1—like, partial	Nucleus Cytoplasm	cellular response to heat response to multiple stresses
1212	213	A0A178VKK2	Glyceraldehyde-3-phosphate dehydrogenase		glucose metabolic process
0105	200	O65396	Glycine cleavage T-protein family	Mitochondrion	glycine decarboxylation via glycine cleavage system response to cadmium ion
1210	137	P25856	Glyceraldehyde 3-phosphate dehydrogenase A subunit	Chloroplast	glucose metabolic process response to cold
0103	82	F4KDZ4	Peroxisomal NAD-malate dehydrogenase 2	Peroxisome	carbohydrate metabolic process
0508	99	P25697	Phosphoribulokinase, chloroplastic	Chloroplast	defense response to bacterium response to cold
**Stem**					
8008	40	P25697	Phosphoribulokinase, chloroplastic	Chloroplast	Defense response to bacteria Response to cold
2301	115	P06525	Alcohol dehydrogenase class-P	Cytoplasm	Response to multiple abiotic stresses
6001	47	P31265	translationally controlled tumor protein-like protein	Cytosol	Auxin homeostasis Response to multiple stresses
4803	481	O23654	vacuolar ATP synthase subunit A	Vacuole	ATP hydrolysis coupled proton transport Response to salt stress

## Data Availability

The data presented in this study are available on request from the corresponding author.

## Supplementary Materials

The following supporting information can be downloaded at: https://www.mdpi.com/article/10.3390/plants11212904/s1, Figure S1: Representative 2D gel of proteins from leaves of control *Arabidopsis thaliana* plant; Figure S2: Representative 2D gel of proteins from leaves of *Cuscuta australis*-parasitized *Arabidopsis thaliana* plant; Figure S3: Representative 2D gel of proteins from stem of control *Arabidopsis thaliana* plant; Figure S4: Representative 2D gel of proteins from stem of *Cuscuta australis*-parasitized *Arabidopsis thaliana* plant.
