# Supplementary figures and images for: Cuscuta australis Parasitism-Induced Changes in the Proteome and Photosynthetic Parameters of Arabidopsis thaliana
Source: Plants (Basel). 2022 Oct 28;11(21):2904. doi: 10.3390/plants11212904 (PMC9656692; doi:10.3390/plants11212904)

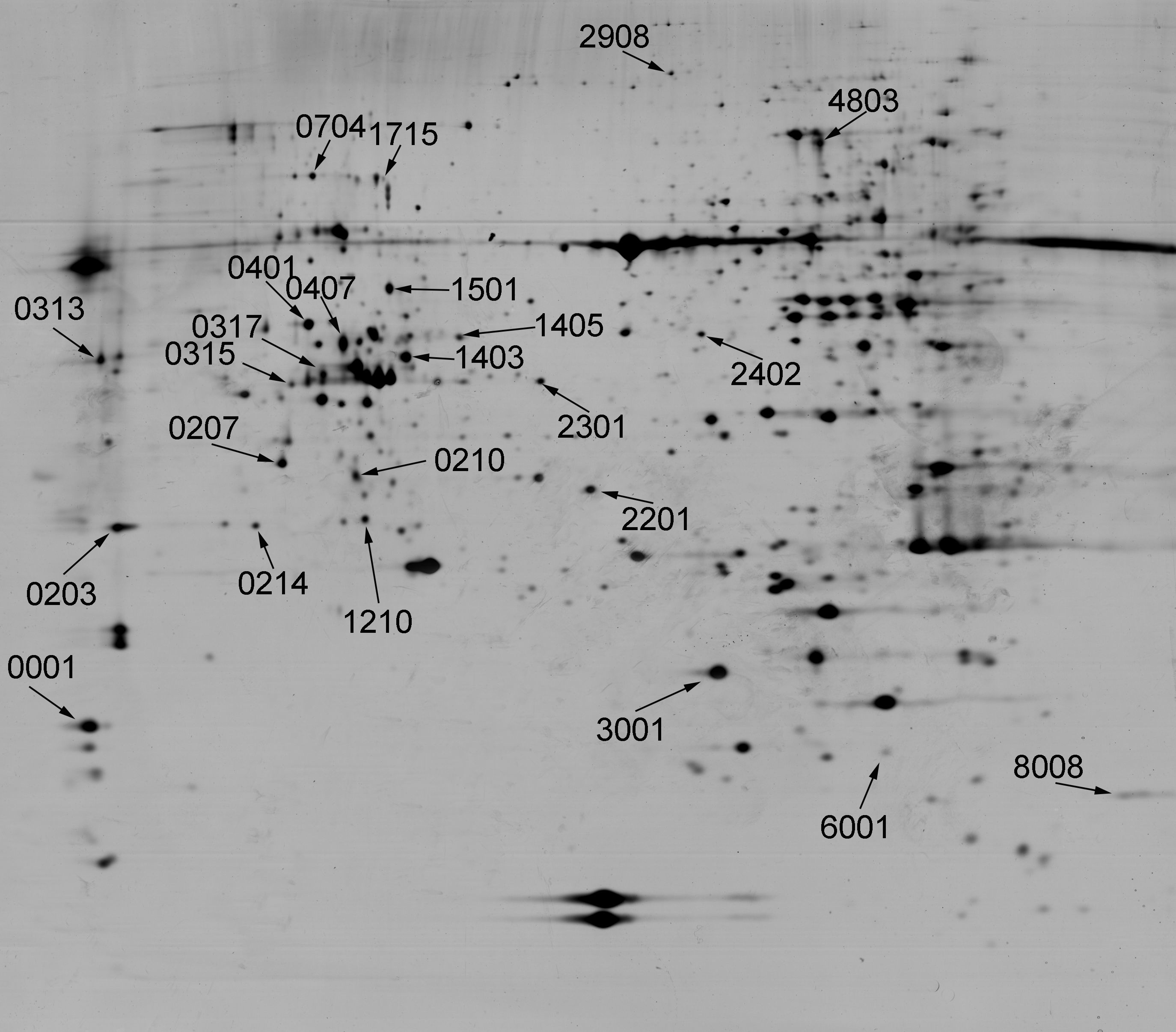

Supplement: Supplementary file 1 [file plants-11-02904-s001.zip › Figure_S4_rev.jpg]

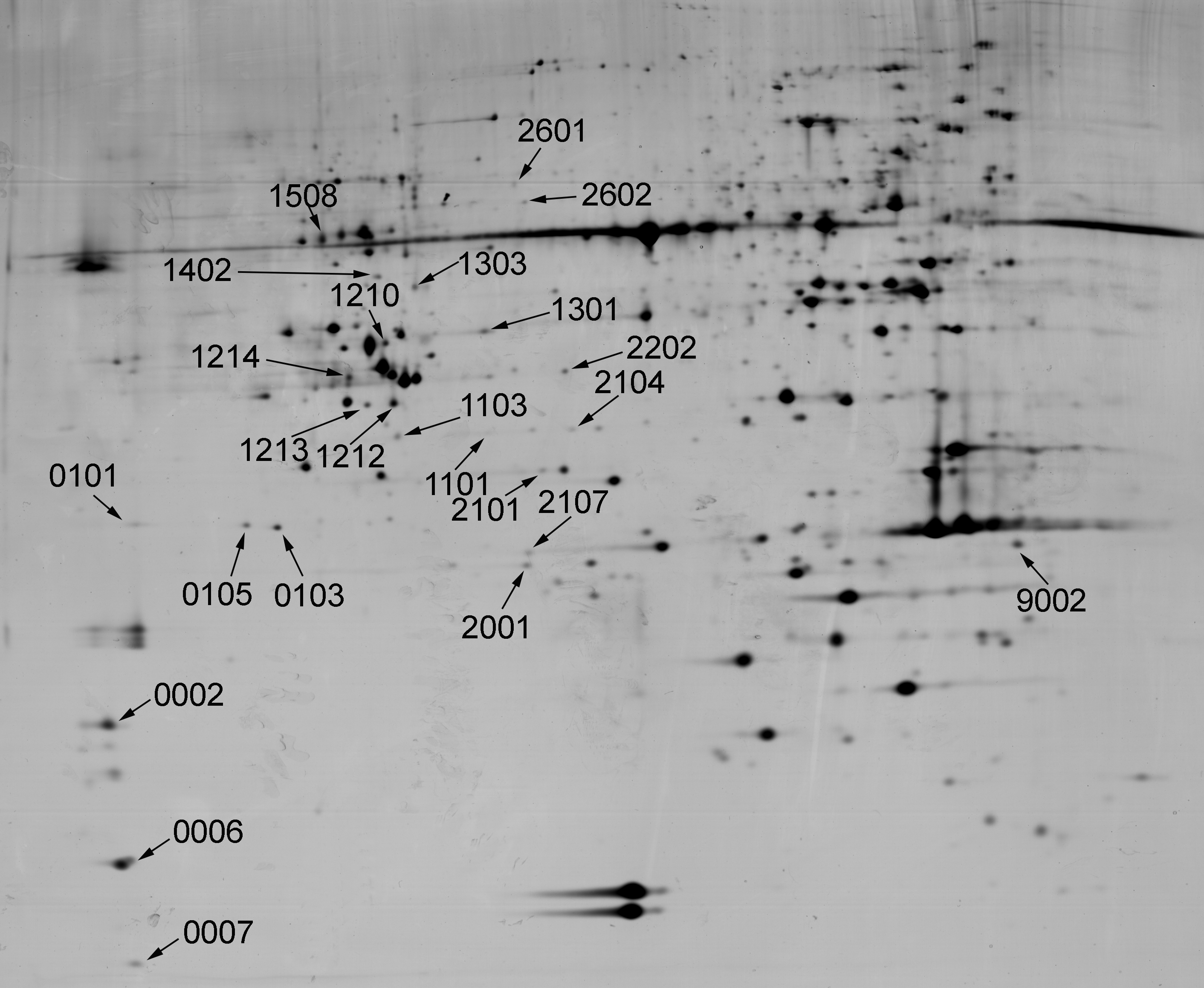

Supplement: Supplementary file 1 [file plants-11-02904-s001.zip › Figure_S1_rev.jpg]

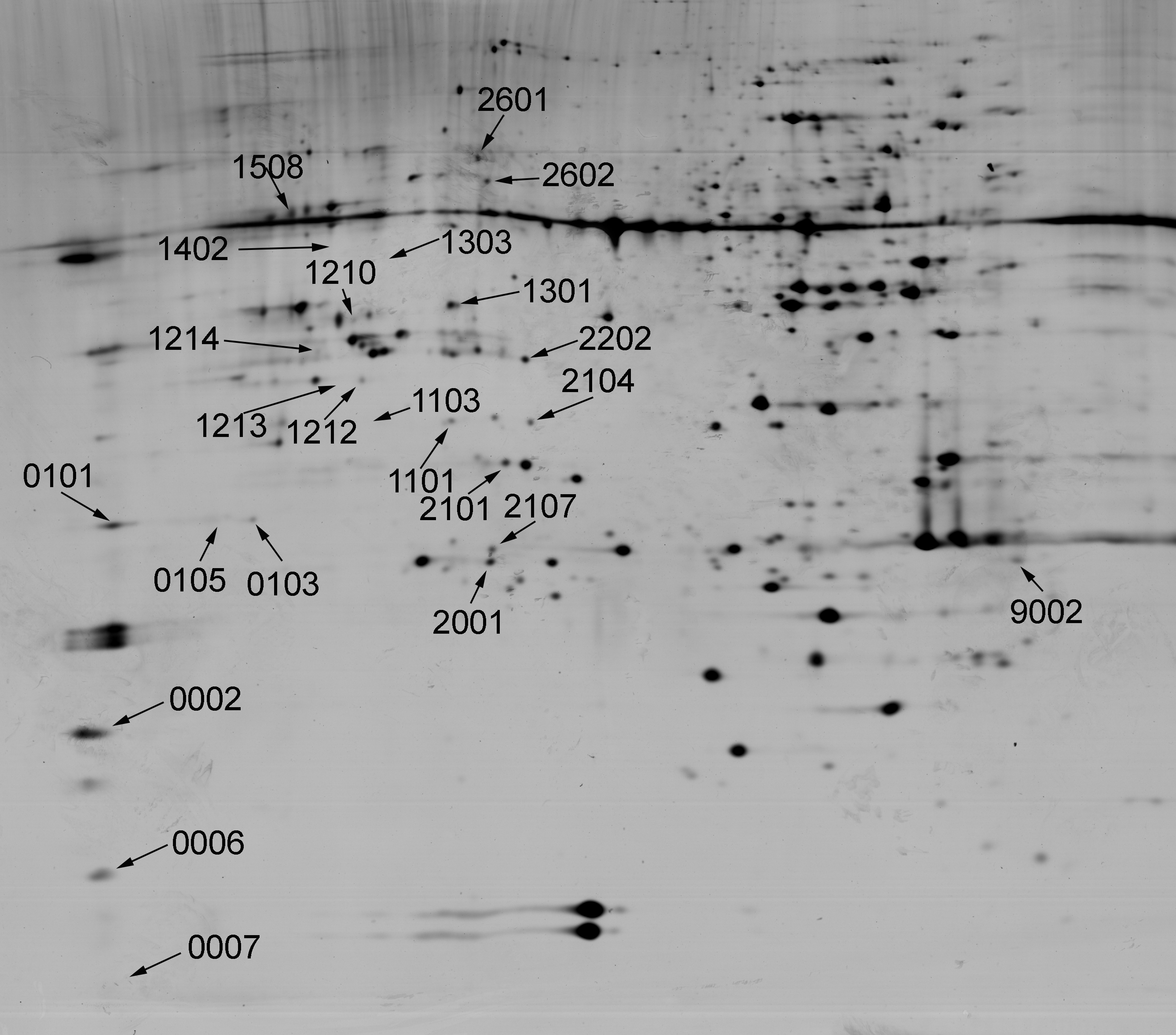

Supplement: Supplementary file 1 [file plants-11-02904-s001.zip › Figure_S2_rev.jpg]

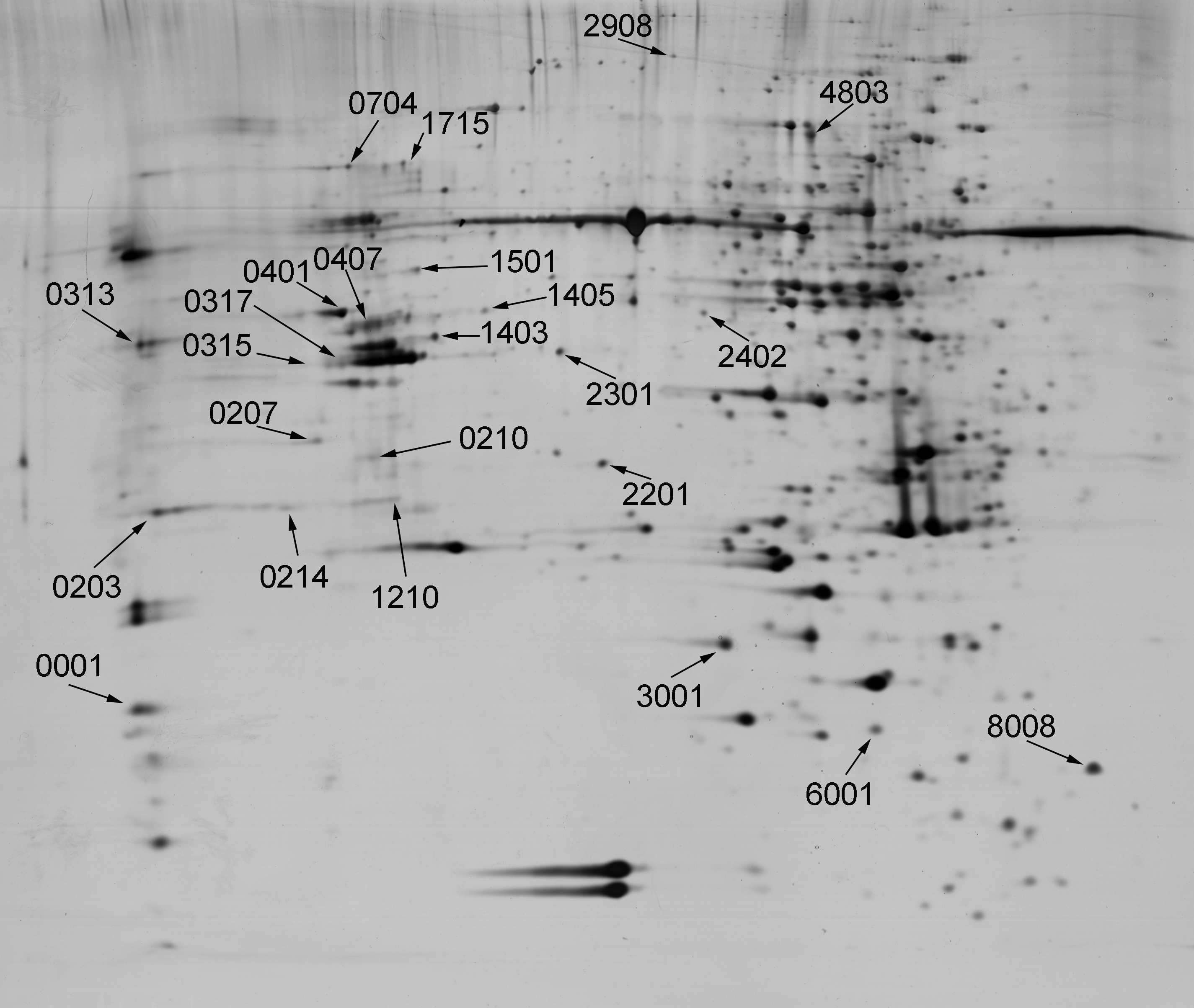

Supplement: Supplementary file 1 [file plants-11-02904-s001.zip › Figure_S3_rev.jpg]
